# A review on the role of PTENP1 in human disorders with an especial focus on tumor suppressor role of this lncRNA

**DOI:** 10.1186/s12935-022-02625-8

**Published:** 2022-06-02

**Authors:** Soudeh Ghafouri-Fard, Tayyebeh Khoshbakht, Bashdar Mahmud Hussen, Mohammad Taheri, Nader Akbari Dilmaghani

**Affiliations:** 1 Department of Medical Genetics, School of Medicine, Shahid Beheshti University of Medical Sciences, Tehran, Iran; 2 Phytochemistry Research Center, Shahid Beheshti University of Medical Sciences, Tehran, Iran; 3 Department of Pharmacognosy, College of Pharmacy, Hawler Medical University, Kurdistan Region, Erbil, Iraq; 4 Center of Research and Strategic Studies, Lebanese French University, Erbil, Kurdistan Region Iraq; 5 Urology and Nephrology Research Center, Shahid Beheshti University of Medical Sciences, Tehran, Iran; 6 Institute of Human Genetics, Jena University Hospital, Jena, Germany; 7 Skull Base Research Center, Loghman Hakim Hospital, Shahid Beheshti University of Medical Sciences, Tehran, Iran

**Keywords:** PTENP1, cancer, Biomarker, Expression

## Abstract

PTENP1 is a long non-coding RNA which has been regarded as a pseudogene of the *PTEN* tumor suppressor gene. However, it has been shown to be a biologically active transcript that can function as a competing endogenous RNA and enhance expression of PTEN protein. This lncRNA has two transcripts, namely PTENP1-202 and PTENP1-202 with sizes of 3996 and 1215 bps, respectively. PTENP1 acts as a sponge for some PETN-targeting miRNAs, such as miR-17, miR-20a, miR-19b, miR-106b, miR-200c, miR-193a-3p, miR-499-5p and miR-214. Besides, it can affect miR-20a/PDCD4, miR-27a-3p/EGR1, miR-17‐5p/SOCS6 and miR-19b/TSC1 axes. This long non-coding RNA participates in the pathoetiology of several types of cancers as well as non-malignant conditions such as alcohol-induced osteopenia, insulin resistance, osteoporosis, sepsis-associated cardiac dysfunction and spinal cord injury. In the current review, we elucidate the role of PTENP1 in human disorders, particularly malignant conditions based on evidence acquired from cell line assays, animal studies and investigations on human samples.

## Introduction

Long non-coding RNAs (lncRNAs) are a group of RNAs with sizes longer than 200 nucleotides, several shared features with mRNAs, the ability to regulate gene expression and lack of significant open reading frames. This novel group of epigenetic regulators mainly resides in the nucleus where they affect histone or DNA modification, chiefly methylation and acetylation [1]. Through influencing alternative splicing, cell differentiation, and cell cycle transition, lncRNAs contribute in the evolution of many diseases [2–4]. Moreover, lncRNAs can affect the organization and function of nuclear bodies, modify the stability and expression of cytoplasmic mRNAs and regulate activity of signaling pathways [5]. Functions and contribution of several lncRNAs in human diseases have been reviewed [6–8].

Phosphatase and Tensin Homolog Pseudogene 1 (PTENP1) is an example of lncRNAs which has been regarded as a pseudogene of the *PTEN* tumor suppressor gene. However, it has been shown to be a biologically active transcript that can function as a competing endogenous RNA (ceRNA) and enhance expression of PTEN protein [9]. In fact, PTENP1 exerts a growth-suppressive effect through obstructing the binding of miRNAs to the 3′ UTR of PTEN and protecting it from degradation [9].

The gene coding this lncRNA is located on chromosome 9: 33,673,504−33,677,499 reverse strand. This lncRNA has two transcripts, namely PTENP1-202 and PTENP1-202 with sizes of 3996 and 1215 bps, respectively (https://asia.ensembl.org/Homo_sapiens/Gene/Summary?g=ENSG00000237984;r=9:33673504-33677499). In the current review, we elucidate the function of PTENP1 in human disorders, particularly malignant conditions based on evidence obtained from cell line assays, animal studies and investigations on human samples.

## Cell line studies

An in vitro experiment in HL-60 promyeoloblastic cells infected with the pCDH1-PTENP1 vectors has shown up-regulation of both PTENP1 and PTEN mRNA levels. However, protein levels of PTEN have not been affected by this intervention. Authors have suggested that PTENP1 can affect PTEN expression at mRNA level [10].

In addition to hematopoietic cells, PTENP1 can affect malignant properties of cell lines originated from solid tumors. Normal cells can secret PTENP1 in their exosomes. Exosome-mediated transmission of this lncRNA to bladder cancer cells could inhibit the malignant features in these cells through induction of cell apoptosis and reduction of invasion and migration abilities of bladder cancer cells. Functionally, exosomal PTENP1 could increase PTEN expression through sponging miR-17 [11]. The PTENP1/miR-20a/PTEN molecular route has been shown to affect malignant behavior of bladder cancer cells. While up-regulation of miR-20a could promote proliferation and migration of T24 cells, PDCD4 over-expression could exert the opposite effects [12].

**Fig. 1:**
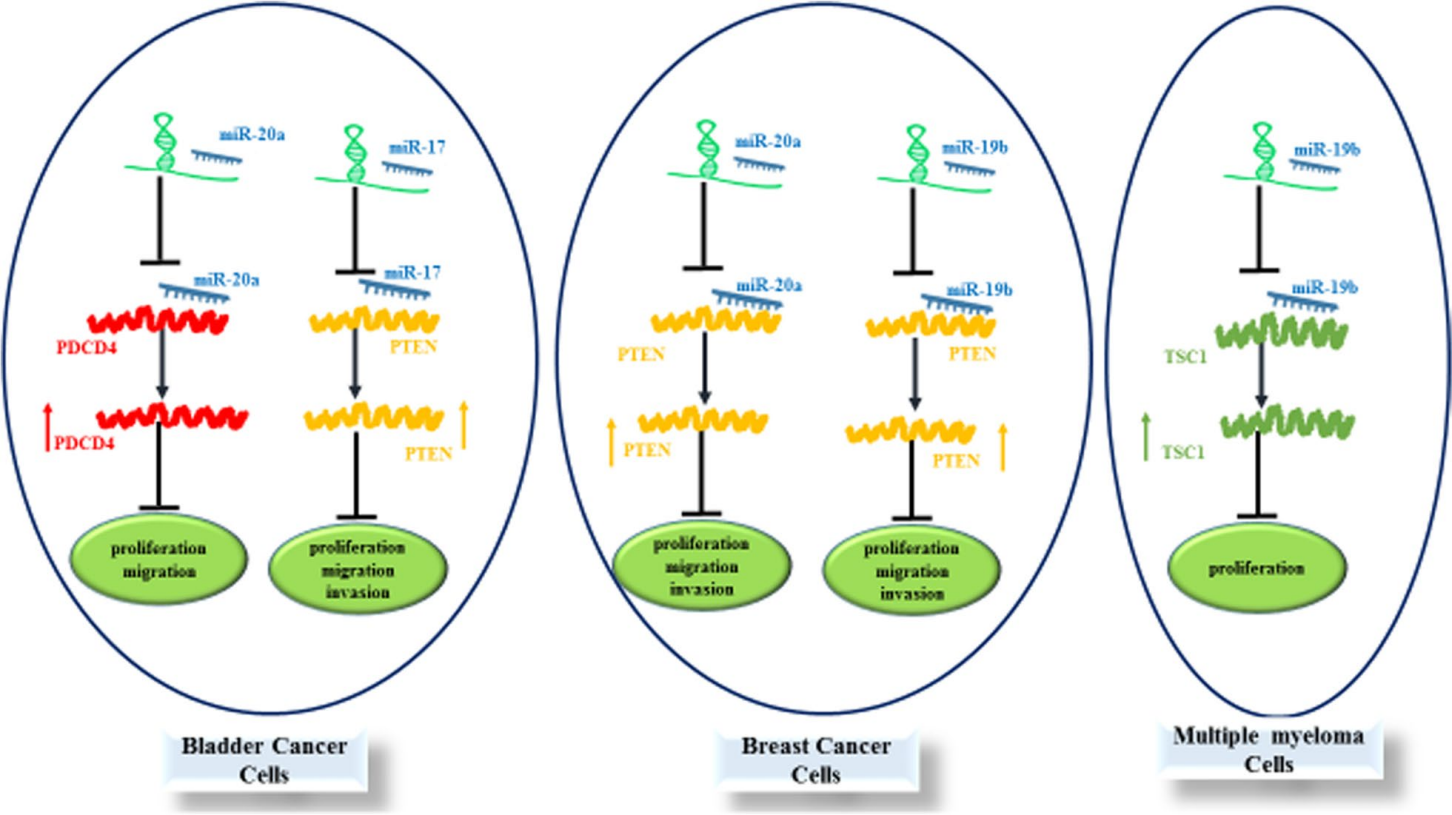
Depicts the roles of PTENP1 in bladder cancer, breast cancer and multiple myeloma

Expression levels of PTENP1 have also been assessed in breast cancer cells. PTENP1 has also been shown to influence proliferation, invasive properties and resistance of breast cancer cells to Adriamycin. These effects are most probably mediated through sponging miR-20a and further regulating expression of PTEN and activity of PI3K/AKT pathway [13]. Moreover, this lncRNA could affect breast cancer pathogenesis through modulation of miR-19b/PTEN axis [14]. PTENP1 could also suppress proliferation and migratory aptitude of breast cancer cells via decreasing expressions of cell cycle regulators cyclin A2 and CDK2 and regulating activity of AKT and MAPK pathways [15]. Finally, the sponging role of PTENP1 on miR-19b has been shown to be implicated in the suppression of proliferation and of breast cancer cells [16] (Fig. 1).

**Fig. 2:**
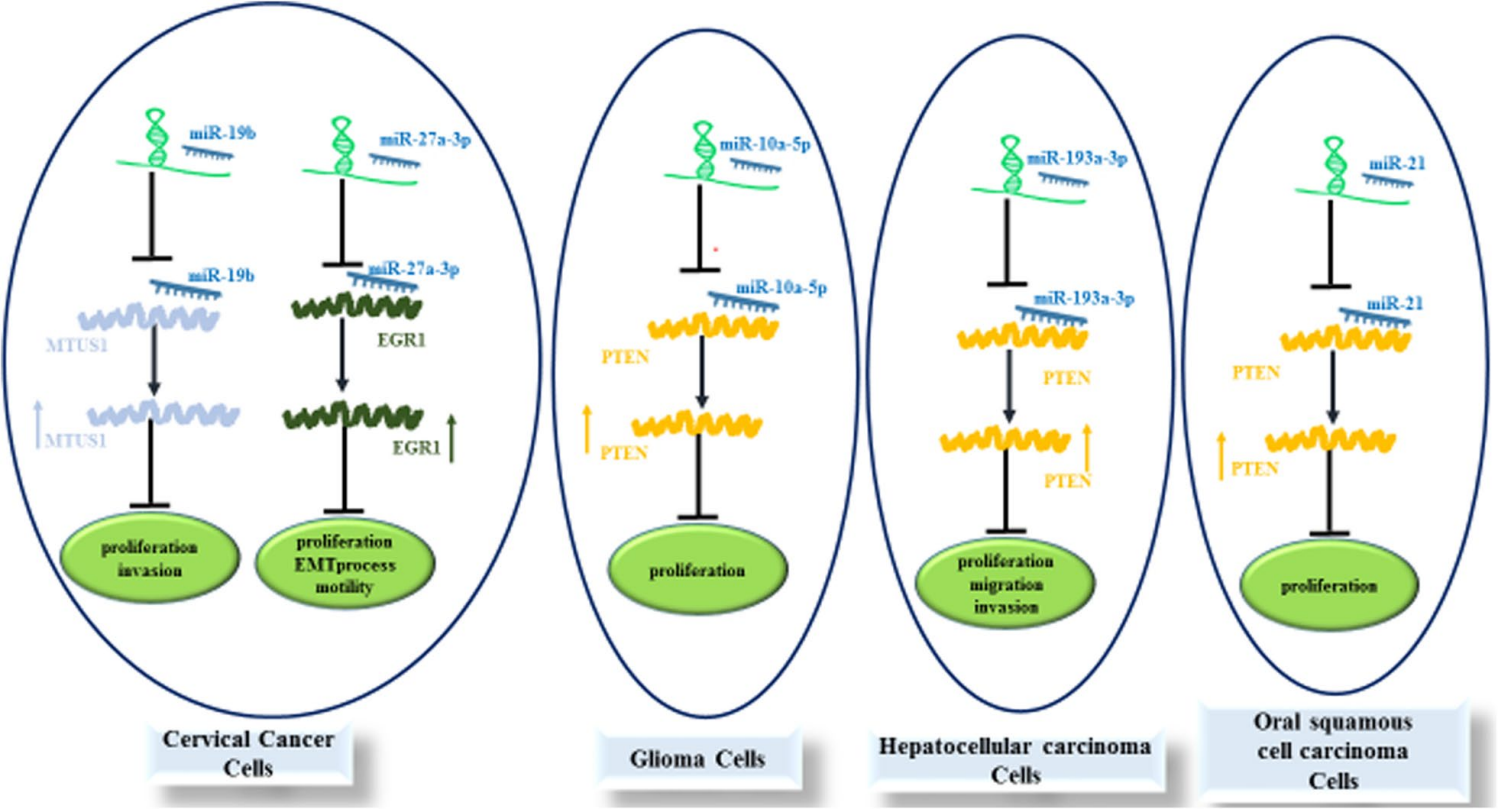
Depicts the tumor suppressor roles of PTENP1 in cervical cancer, glioma, hepatocellular carcinoma and oral squamous cell carcinoma

Similarly, PTENP1 could inhibit progression of cervical cancer through different mechanisms including suppression of miR-106b [17], miR-27a-3p [18] and miR-19b [19]. These miRNAs target PTEN, EGR1 and MTUS1, respectively (Fig. 2).

Figure 1. Summary of the role of PTENP1 in progression of cancers. PTENP1 can serve as molecular sponge for miR-19b, miR-20a and miR-17. Down-regulation of these miRNAs by PTENP1 affects proliferation, migration and invasiveness of cancer cells. Detailed information about the impact of this lncRNA on suppression of carcinogenesis is provided in Table 1.

Figure 2. Summary of the role of PTENP1 in progression of cancers. PTENP1 can serve as molecular sponge for miR-21, miR-10a-5p, miR-19b, miR-27a-3p, miR-193a-3p, miR-19b, miR-20a and miR-17. Down-regulation of these miRNAs by PTENP1 induces anti-tumor effects. Detailed information about the impact of this lncRNA on suppression of carcinogenesis is provided in Table 1.

**Table 1:** Role of PTENP1 in different cancers according to cell line studies

Tumor	Interactions	Cell line	Function	References
Acute leukemia	PTEN	HL-60 cell line and 293T cells	↑↑ PTENP1: ↑ PTEN mRNA level without affecting PTEN protein levels and cell growth	[10]
Bladder cancer	miR-17/PTEN axis	EJ, J82, HEK 293 A	↑↑ PTENP1: ↓ proliferation, migration, invasion, colony formation, ↑ apoptosis	[11]
	miR-20a/PDCD4 axis	Human bladder cancer cell lines J82 and T24, SV-HUC-1	↑↑ miR-20a (a target of PTENP1): ↑ proliferation and migration	[12]
Breast cancer	miR-20a/PTEN axis, PI3K/Akt signaling	MDA-MB-231, T-47D and MCF-7 , mammary epithelium MCF-10 A	↑↑ PTENP1: ↓ proliferation, migration, invasion, colony formation, viability	[13]
	miR-19b/ PTEN axis, p53 and p-AKT	MCF-10 A,BT-20, MCF-7, MDA-MB-231 and T-47D	↑↑ PTENP1: ↓ proliferation, migration, invasion, ↑ apoptosis ↑ p53 and ↓p-AKT	[14]
	AKT and MAPK signaling pathways	MCF7, 293T	↑↑ PTENP1: ↓ proliferation, migration, colony formation, cyclin A2 and CDK2, AKT and MAPK signaling pathways	[15]
	miR-19b/ PTEN axis and PI3K/Akt Pathway	MCF10A, MCF-7 and MDA-MB-231	↑↑ PTENP1: ↓ proliferation, migration, invasion, colony formation, PDK-1, p-PI3K, PI3K, and p-Akt, ↑ apoptosis, PTEN	[16]
Cervical cancer	miR-106b/ PTEN axis	HeLa, SiHa, C33A, CasKi, H8	↑↑ PTENP1: ↓ proliferation, EMT process, ↑ apoptosis	[17]
	miR-27a-3p/ EGR1 axis	C33A, HeLa, ME-180, SiHa, NC104	↑↑ PTENP1: ↓ proliferation, EMT process, motility, ↑ apoptosis	[18]
	miR-19b/ MTUS1 axis	Human normal cervical epithelium cell (HCvEpC) and human CC cell lines, such as Caski, C33A, SiHa and HeLa cells	↑↑ PTENP1: ↓ proliferation and invasion	[19]
Endometrioid endometrial carcinoma	miR-200c/ PTEN axis and PI3K-AKT pathway	RL-952, Ishikawa, HEC-1B, and JEC	17β-estradiol (E2) treatment: ↑ proliferation, migration and invasion, miR-200c levels, phospho-PI3K-AKT pathway genes and ↓ PTEN level ∆ ERα: ↓ effects of E2 on miR-200c and PTEN	[20]
Esophageal carcinoma	miR-17‐5p/ SOCS6 axis, p‐STAT3‐HIF‐1α signal pathway	Eca109, TE-1, HEK‐293T, Het‐1 A	↑↑ PTENP1: ↓ proliferation, vitality, p-STAT3‐HIF‐1α signal pathway	[21]
Gastric cancer	miR-106b, miR-93 and PTEN	GES-1, gastric adenocarcinoma cell line AGS, SGC7901, MGC803 and BGC823	↑↑ PTENP1: ↓ cell growth, migration, and invasion, ↑ apoptosis	[22]
Glioma	p21 and p38 signaling pathway.	SHG44 and U251 human glioma cells	↑↑ PTENP1: ↓ proliferation, migration, and invasion, p38 MAPK signaling pathway, ↑ cell cycle arrest, p21 levels	[23]
	miR-10a-5p/ PTEN axis	Glioma cell line U87	Co-Culture of hUC-MSCs-derived exosomes suppress the proliferation and stimulate the apoptosis of U87 Cells. Exosomes-Mediated Transfer of LncRNA PTENP1 suppresses Cell Growth by Targeting MiR-10a-5p.	[24]
Head and neck squamous cell carcinoma	PTEN	WSU-HN4, HN6, HN13, HN30 and Cal27	↑↑ PTENP1: ↓ cell growth, migration, invasion, colony formation	[25]
Hepatocellular carcinoma	miR-21, TET1/2/3, PTEN	SNU-449, HepG2, Hep3B, Huh7	↑↑ miR-21: ↑ proliferation, invasion, ↓ apoptosis, expression of TET1/2/3, change in methylation and expression of PTENp1, ↓ PTENp1 and PTEN	[26]
	miR-193a-3p/ PTEN axis	Sk-Hep-1 and SMMC-7721	↑↑ PTENP1: ↓ proliferation, migration, invasion, ↑ apoptosis	[27]
	miR-17, miR-19b and miR-20a, PTEN, PHLPP, ULK1, ATG7 and p62, ↓ PI3K/AKT pathway	human hepatocytes (HH) and HCC cell line Mahlavu	↑↑ PTENP1: ↓ proliferation, migration, invasion ↑ autophagy and apoptosis	[28]
Multiple myeloma	miR-19b/ TSC1 axis	OPM2 and KMS-11 cells	↑↑ PTENP1: ↓ miR-19b levels and ↑ proliferation	[29]
Oral squamous cell carcinoma	miR-21/ PTEN, AKT pathways	SCC-25, Cal-27, and HEK 293 cells and ca-8113,	↑↑ PTENP1: ↓ proliferation, ↑ cell cycle arrest	[30]
Renal cell carcinoma	miR21/ PTEN axis	Human renal cell carcinoma cell lines 786-O, ACHN, SN12PM6 and HK-2	↑↑ PTENP1: ↓ proliferation and cell growth, migration, invasion, metastasis, and ↑ sensitivity of ccRCC cells to cisplatin and gemcitabine	[31]

*BC* breast cancer, *ccRCC* clear-cell renal cell carcinoma, ∆ knock-down or deletion

PTENP1 can also affect pathoetiology of non-malignant conditions (Table 2). For instance, it can affect pathogenesis of alcohol-induced osteopenia. Ethanol stimulation has resulted in up-regulation of expression of PTEN and PTENP1 transcripts in a time-dependent mode, leading to up-regulation of PTEN protein levels. Moreover, ethanol could decrease PTEN phosphorylation, representing an upsurge in functional PTEN level. Up-regulation of PTEN could impair downstream Akt/GSK3β/β-catenin signals and osteogenic differentiation of bone mesenchymal stem cells [32]. Moreover, PTENP1 binding to miR-499-5p leads to deficiency in the insulin-signaling pathway, thus participating in insulin resistance [33]. Furthermore, up-regulation of PTENP1 or silencing of miR-214 could inhibit expressions of osteoclast markers and RANKL-induced osteoclast differentiation. These interventions could also inhibit phosphorylation of PI3K and AKT, nuclear transport of p65, destruction of IκBα and NFATc1 expression. On the other hand, PTENP1 silencing has enhanced osteoclast differentiation. Taken together, PTENP1 acts as a sponge for miR-214 to escalate expression of PTEN and suppress osteoclast differentiation. This mode of action attenuates osteoporosis through inhibition of PI3K/AKT/NF-κB signaling [34].

**Table 2:** Role of PTENP1 in different non-malignant conditions according to cell line studies

Disorders	Interaction	Cell line	Function	References
Alcohol-induced osteopenia	PTEN and Akt/GSK3β/β-catenin signaling	Human BMSCs (hBMSCs)	Ethanol treatment: ↑ PTEN and PTENP1 levels and ↓ Akt/GSK3β/β-catenin signaling ∆ PTEN: ↓ ethanol-induced suppression of bone formation and antiosteogenic effect of ethanol	[32]
Insulin resistance	miR-499-5p/ PTEN axis	Murine liver cell line NCTC1469	↑↑ PTEN: ↓ Akt/GSK activation and glycogen synthesis	[33]
Osteoporosis	miR-214/ PTEN axis, 3 K/AKT/NF-kB signaling pathway	RAW 264.7 macrophages	↑↑ PTENP1: ↓ ANKL- induced osteoclast differentiation BY inhibiting 3 K/AKT/NF-kB signaling pathway	[34]
Sepsis-associated cardiac dysfunction	miR-106b-5p	H9C2	Matrine administration: ↓ expression of PTENP1 and inflammation, ↑ H9C2 viability	[35]
Spinal cord injury	miR-21, miR-19b and PTEN	SH-SY5Y and U251 cells	∆ PTENP1: ↑ apoptosis, miR-21, miR-19b and ↓ cell viability	[36]

## Animal studies

Impact of PTENP1 up-regulation and exosomal PTENP1 on growth of tumors has been investigated *in vivo*. Authors have injected EJ cells with PTENP1-expressing vectors as well as PTENP1-containing exosomes into nude mice. The results of conducted experiments have indicated that up-regulation of PTENP1 can decrease tumor weight and burden. Moreover, PTENP1-containing exosomes could attenuate tumor size and weight. Besides, over-expression of this lncRNA could reduce Ki67 expression in tumors [11]. Other studies in esophageal carcinoma, head and neck squamous cell carcinoma, hepatocellular cancer and oral squamous cell carcinoma have confirmed the impact of PTENP1 up-regulation on attenuation of tumor growth (Table 3). In animal models of renal cell carcinoma, up-regulation of this lncRNA has enhanced sensitivity to cisplatin and gemcitabine [31].

Animal models have also been used to evaluate the impact of PTENP1 in insulin resistance. An experiment in db/db mice and high fat diet-fed mice has shown up-regulation of PTENP1. Moreover, up-regulation of PTENP1 has led to impairment in activation of Akt/GSK and production of glycogen, while suppression of this lncRNA has enhanced activity of Akt/GSK and increased glycogen content [33]. In an in vivo study, it has shown that the effect of matrine on improvement of cardiac function and attenuation of the inflammatory responses is mediated through down-regulation of PTENP1 expression and up-regulation of miR-106b-5p levels [35].

**Table 3:** PTENP1 role in different disorders based on animal studies

Tumor/ disease type	Animal models	Results	References
Bladder cancer	5 week-old male nude mice injected with EJ cell lines	↑↑ PTENP1: ↓ tumor weight, tumor volume and tumor size	[11]
Esophageal carcinoma	4 week-old male nude mice injected with Eca109 cells transfected with PTENP1 3′UTR or NC	↑↑ PTENP1: ↓ tumorigenesis	[21]
Head and neck squamous cell carcinoma	4-week-old male nude mice	↑↑ PTENP1: ↓ tumorigenesis	[25]
Hepatocellular carcinoma	4 week-old BALB/c nude mouse	∆ miR-21: ↓ tumor growth and size, ↑ PTEN, PTENp1, TET1, TET2 and TET3	[26]
	4 week-old male immune-deficient nude mice (BALB/c-nu)	↑↑ PTENP1: ↓ tumor weight and tumor volume	[27]
	6-8-weeks-old BALB/c nude mic were injected with Mahlavu cells	↑↑ PTENP1: ↓ tumor growth, intratumoral cell proliferation, and angiogenesis, ↑ apoptosis, autophagy	[28]
Oral squamous cell carcinoma	5 -week-old female BALB/C nude mice mice were injected with Tca-8113 cells transfected with LV-miR-21 plus LV-PTEN and LV-PTENp1	↑↑ PTENP1: ↓ tumorigenesis	[30]
Renal cell carcinoma	Nude mice were injected with ACHN cells transfected with vector control or PTENP1	↑↑ PTENP1: ↑ sensitivity of ccRCC cells to cisplatin and gemcitabine	[31]
Alcohol-induced osteopenia	40 8-week-old male specific SPF and SD rats	∆ PTEN: ↓ ethanol-induced osteopenia	[32]
Insulin resistance	5 db/db mice and 5 age-matched wild-type (WT) mice	↑↑ PTENP1: ↑ hepatic insulin resistance	[33]
Osteoporosis	8-week-old female C57BL/6 mice	Levels of PTENP1 and PTEN were down-regulated in CS-F- and RANKL-induced bone marrow mononuclear cell.	[34]
Spinal cord injury	Rats in sham group and SCI, SCI + exosomes, and SCI + exosomes + PTENP1-shRNA groups	Treatment with exosomes + PTENP1-shRNA: ↓ PTEN expression PTENP1 participates in the recovery of SCI through regulation of miR-19b and miR-21.	[36]

*∆* knock-down or deletion, *ccRCC* clear-cell renal cell carcinoma, *SPF* specific pathogen-free, *SD* Sprague–Dawley

## Clinical studies

Expression of PTEN and PTENP1 mRNAs has been demonstrated to be lower in bone marrow samples of AML patients compared to healthy subjects. Moreover, expressions of these transcripts have been positively correlated. However, when AML patients have been classified based on the prognostic classification of 2011 NCCN, authors have detected no remarkable difference in the expression of PTENP1 among subgroups [10].

Expression of PTENP1 has also been shown to be diminished in bladder cancer tissues as well as exosomes extracted from plasma samples of these patients. In fact, this lncRNA has been found to be principally carried by exosomes. Exosomal levels of PTENP1 have the potential to discriminate bladder cancer patients from healthy subjects with area under receiver characteristic curve of 0.743. Thus, exosomal PTENP1 has been recommended as a putative marker for diagnostic purposes in bladder cancer [11]. In bladder cancer cells, PTENP1 target miR-20a has been shown to be up-regulated, while PDCD4 has been down-regulated [12].

In breast cancer, cervical cancer, head and neck squamous cell carcinoma, hepatocellular carcinoma and oral squamous cell carcinoma, down-regulation of PTENP1 has been linked with poor survival of patients (Table 4). Moreover, down-regulation of this lncRNA has been correlated with advanced histological grade and TNM stage, deep infiltration depth, and lymph node metastasis in cancer patients.

Association between a number of tag single nucleotide polymorphisms within PTENP1, including rs7853346 C > G, rs865005 C > T, and rs10971638 G > A and susceptibility to gastric cancer has been assessed in a Chinese population. Results have shown association between rs7853346 G allele and lower risk of gastric cancer. This association has been stronger in patients aged more than 60 years, non-smokers, non-drinkers, and those without family history of gastric cancer. Notably, expression assays have shown higher levels of PTENP1 in carriers of rs7853346 CG/GG genotype [37].

PTENP1 has also been shown to be down-regulated in osteoporosis patients, parallel with up-regulation of miR-214 [34].

**Table 4:** Dysregulation of PTENP1 in clinical samples

Tumor/ disease type	Numbers of clinical samples	Expression (Tumor vs. normal)	Kaplan-Meier analysis	Polymorphism in PTENP1 associated with Tumor/ disease	Multivariate/ univariate cox regression	Clinicopathologic characteristics of patients	References
Acute leukemia	138 AL patients and 15 healthy controls	Downregulated					[10]
Bladder cancer	Plasma samples from 50 patients with bladder cancer and 60 normal subjects 20 pairs of tumor tissues and ANTs	Downregulated				High clinical grade	[11]
	60 pairs of tumor tissues and ANTs	Upregulation of miR-20a (a target of PTENP1)					[12]
Breast cancer	52 pairs of tumor tissues and ANTs	Downregulated	Poorer OS			Advanced BC stages	[13]
	65 pairs of tumor tissues and ANTs	Downregulated					[14]
	20 pairs of tumor tissues and ANTs	Downregulated					[16]
Cervical cancer	54 pairs of tumor tissues and ANTs	Downregulated				FIGO stage and the lymph node metastasis	[17]
	88 pairs of tumor tissues and ANTs	Downregulated	Poorer OS			Advanced stage, FIGO stage, tumor size and lymph node metastasis	[18]
	56 pairs of tumor tissues and ANTs	Downregulated	Poorer OS			Advanced FIGO stage, metastasis and recurrence	[19]
Endometrioid endometrial carcinoma	40 pairs of tumor tissues and ANTs GEO database and TGCA database	Downregulated					[20]
Esophageal carcinoma	GEO database (GSE20347): 17 pairs of tumor tissues and ANTs	Downregulated					[21]
	93 ESCC patients	Downregulated	Poorer OS		TNM stage and PTENP1 expression were found to be independent factors that influence the OS of patients after radical esophagectomy.	Histological grade, more advanced TNM stage, deep infiltration depth, and lymph node metastasis	
Gastric cancer	768 GC patients and 768 healthy controls	Downregulated		Patients who had rs7853346 G allele showed a remarkably decreased risk of GC in comparison with those carrying C allele. Samples with rs7853346 CG/GG genotype showed high PTENP1 mRNA expression levels than those with CC genotype.			[37]
Gastric cancer	36 pairs of tumor tissues and ANTs	Downregulated				Tumor size, clinic stage and invasion depth	[22]
Glioma	23 gliomas tissue samples	Downregulated					[23]
	279 glioma patients	Downregulated		Downregulated in patients carrying the CG&GG genotypes of rs7853346 compared with patients carrying the CC genotype of rs7853346			[38]
Head and neck squamous cell carcinoma	57 HNSCC tissues and 27 ANTs	Downregulated	Poorer OS or DFS		PTENP1 level was found to be an independent predictor of the OS and DFS in patients.	History of alcohol use	[25]
Hepatocellular carcinoma	48 pairs of tumor tissues and ANTs	Downregulated	Poorer OS			Tumor size and TNM stage	[27]
	129 patients with HCC, 49 patients with liver cirrhosis, 27 patients with chronic HBV, and 93 normal subjects	Downregulated in HCC than in control groups					[39]
Multiple myeloma	43 multiple myeloma patients and 35 healthy controls	Upregulated		Samples with CC genotype showed higher levels of PTENP1 and TSC1 mRNA, and lower level of miR-19b compared to the CG and GG groups. G allele of rs7853346 polymorphism induces the proliferation of cancer stem cells.			[29]
Oral squamous cell carcinoma	62 pairs of tumor tissues and ANTs	Downregulated	Poorer OS			pT-stage and clinical stage	[30]
	342 OSCC patients and 711 healthy controls 20 pairs of tumor tissues and ANTs			rs7853346 strongly reduced OSCC risk.	rs7853346 strongly decreased OSCC risk with gender, age, smoking and drinking condition adjusted.		[40]
Renal cell carcinoma	40 pairs of tumor tissues and ANTs	Downregulated					[31]
Osteoporosis	30 postmenopausal females with osteoporosis and 15 premenopausal females with arthritis (as controls)	Downregulated					[34]

*ANTs* adjacent non-cancerous tissues, *OS* overall survival, *TNM* tumor-node‐metastasis, *HCC* hepatocellular carcinoma, *ESCC* esophageal squamous cell carcinoma, *HNSCC* head and neck squamous cell carcinoma, *DFS* disease-free survival, *AL* acute leukemia

## Discussion

PTENP1 is an lncRNA which primarily functions as a ceRNA to enhance expression of PTEN. This lncRNA acts as a sponge for some PETN-targeting miRNAs, such as miR-17, miR-20a, miR-19b, miR-106b, miR-200c, miR-193a-3p, miR-499-5p and miR-214. Besides, it can serve as a molecular sponge for other miRNAs such as miR-20a, miR-27a-3p, miR-17‐5p and miR-19b to influence expressions of PDCD4, EGR1, SOCS6 and TSC1, respectively.

The role of PTENP1 has been mostly evaluated in the pathoetiology of cancer. In this context, the results of *in vitro*, *in vivo* and clinical studies have been consistent. This lncRNA is regarded as a tumor suppressor lncRNA in all cancers except for multiple myeloma.

In addition, a number of investigations have shown its influence on development of non-malignant conditions such as alcohol-induced osteopenia, insulin resistance, osteoporosis, sepsis-associated cardiac dysfunction and spinal cord injury.

As an lncRNAs secreted in the exosomes, it has the potential to be used as a biomarker for early detection of cancers. This application has been evaluated in the context of bladder cancer. However, further studies in other cancers are needed to appraise the potential of PTENP1 in diagnostic purposes.

Although forced up-regulation of PTENP1 in cancer cell lines using different vectors could attenuate *in vitro* cancer cell proliferation and *in vivo* tumor growth, this field of study is still in its initial phases, needing further evaluations in animal models particularly focusing on bioavailability and biosafety issues. Additionally, a comprehensive evaluation of PTENP1 targets and related signaling pathways is necessary to avoid unwanted side effects.

Since up-regulation of PTENP1 can also enhance the cytotoxic effects of chemotherapeutic agents on cancer cells, therapies aimed at over-expression of this lncRNA are potential ways for combating chemoresistance.

## Conclusions

Association between PTENP1 polymorphisms and susceptibility to cancer has been evaluated in Chinese gastric cancer patients. Additional studies in other types of cancers in different populations are needed to find the influence of genetic variants in this lncRNA on cancer risk.

Taken together, PTENP1 is an important modulator of cancer progression which not only increases expression of the important tumor suppressor PTEN, but also affects expression of other cancer-related genes such as those regulating cell cycle progression. Thus, this lncRNA represent a promising target for design of novel anti-cancer therapies.

## Data Availability

The analyzed data sets generated during the study are available from the corresponding author on reasonable request.

## References

[1] Zhang X, Wang W, Zhu W, Dong J, Cheng Y, Yin Z (2019). Mechanisms and functions of long non-coding RNAs at multiple regulatory levels. Int J Mol Sci.

[2] Fatica A, Bozzoni I (2014). Long non-coding RNAs: new players in cell differentiation and development. Nat Rev Genet.

[3] Kitagawa M, Kitagawa K, Kotake Y, Niida H, Ohhata T (2013). Cell cycle regulation by long non-coding RNAs. Cell Mol Life Sci.

[4] Yang G, Lu X, Yuan L (2014). LncRNA: a link between RNA and cancer. Biochimica et Biophysica Acta (BBA).

[5] Statello L, Guo C-J, Chen L-L, Huarte M (2021). Gene regulation by long non-coding RNAs and its biological functions. Nat Rev Mol Cell Biol.

[6] Ghafouri-Fard S, Khoshbakht T, Hussen BM, Taheri M, Mokhtari M (2021). A review on the role of AFAP1-AS1 in the pathoetiology of cancer. Front Oncol.

[7] Ghafouri-Fard S, Khoshbakht T, Taheri M, Jamali E (2021). A concise review on the role of CircPVT1 in tumorigenesis, drug sensitivity, and cancer prognosis. Front Oncol.

[8] Ghafouri-Fard S, Khoshbakht T, Taheri M, Ebrahimzadeh K (2021). A review on the carcinogenic roles of DSCAM-AS1. Front Cell Develop Biol.

[9] Wang Z (2018). Antisense RNA and cancer. Cancer and noncoding RNAs.

[10] Wang C, Huai L, Zhang C, Jia Y, Li Q, Chen Y (2012). Study on expression of PTEN gene and its pseudogene PTENP1 in acute leukemia and correlation between them. Zhonghua xue ye xue za zhi Zhonghua xueyexue zazhi..

[11] Zheng R, Du M, Wang X, Xu W, Liang J, Wang W (2018). Exosome–transmitted long non-coding RNA PTENP1 suppresses bladder cancer progression. Mol Cancer.

[12] Zhong X, Wang L, Yan X, Yang X, Xiu H, Zhao M (2020). MiR-20a acted as a ceRNA of lncRNA PTENPL and promoted bladder cancer cell proliferation and migration by regulating PDCD4. Eur Rev Med Pharmacol Sci.

[13] Gao X, Qin T, Mao J, Zhang J, Fan S, Lu Y (2019). PTENP1/miR-20a/PTEN axis contributes to breast cancer progression by regulating PTEN via PI3K/AKT pathway. J Exp Clin Cancer Res.

[14] Li R, Guo L, Huang G, Luo W (2017). PTENP1 acts as a ceRNA to regulate PTEN by sponging miR-19b and explores the biological role of PTENP1 in breast cancer. Cancer Gene Ther.

[15] Chen S, Wang Y, Zhang J-H, Xia Q-J, Sun Q, Li Z-K (2017). Long non-coding RNA PTENP1 inhibits proliferation and migration of breast cancer cells via AKT and MAPK signaling pathways. Oncol Lett.

[16] Shi X, Tang X, Su L (2018). Overexpression of long noncoding RNA PTENP1 inhibits cell proliferation and migration via suppression of miR-19b in breast cancer cells. Oncol Res.

[17] Fan Y, Sheng W, Meng Y, Cao Y, Li R (2020). LncRNA PTENP1 inhibits cervical cancer progression by suppressing miR-106b. Artif cells Nanomed Biotechnol.

[18] Wu C, Wang F, Tan L (2020). Role and the molecular mechanism of lncRNA PTENP1 in regulating the proliferation and invasion of cervical cancer cells. Gene Ther.

[19] Ou L, Xiang T, Hao X, Wang D, Zeng Q (2020). Reduced long non-coding RNA PTENP1 contributed to proliferation and invasion via miR-19b/MTUS1 axis in patients with cervical cancer. Eur Rev Med Pharmacol Sci.

[20] Chen R, Zhang M, Liu W, Chen H, Cai T, Xiong H (2018). Estrogen affects the negative feedback loop of PTENP1-miR200c to inhibit PTEN expression in the development of endometrioid endometrial carcinoma. Cell Death Dis.

[21] Gong T, Zheng S, Huang S, Fu S, Zhang X, Pan S (2017). PTENP1 inhibits the growth of esophageal squamous cell carcinoma by regulating SOCS6 expression and correlates with disease prognosis. Mol Carcinog.

[22] Zhang R, Guo Y, Ma Z, Ma G, Xue Q, Li F (2017). Long non-coding RNA PTENP1 functions as a ceRNA to modulate PTEN level by decoying miR-106b and miR-93 in gastric cancer. Oncotarget.

[23] Hu S, Xu L, Li L, Luo D, Zhao H, Li D (2019). Overexpression of lncRNA PTENP1 suppresses glioma cell proliferation and metastasis in vitro. Onco Targets  Ther.

[24] Hao S, Ma H, Niu Z, Sun S, Zou Y, Xia H (2019). hUC-MSCs secreted exosomes inhibit the glioma cell progression through PTENP1/miR-10a-5p/PTEN pathway. Eur Rev Med Pharmacol Sci.

[25] Liu J, Xing Y, Xu L, Chen W, Cao W, Zhang C (2017). Decreased expression of pseudogene PTENP1 promotes malignant behaviours and is associated with the poor survival of patients with HNSCC. Sci Rep.

[26] Cao L-q, Yang X-w, Chen Y-b, Zhang D-w, Jiang X-F, Xue P (2019). Exosomal miR-21 regulates the TETs/PTENp1/PTEN pathway to promote hepatocellular carcinoma growth. Mol Cancer.

[27] Qian Y-Y, Li K, Liu Q-Y, Liu Z-S (2017). Long non-coding RNA PTENP1 interacts with miR-193a-3p to suppress cell migration and invasion through the PTEN pathway in hepatocellular carcinoma. Oncotarget.

[28] Chen C-L, Tseng Y-W, Wu J-C, Chen G-Y, Lin K-C, Hwang S-M (2015). Suppression of hepatocellular carcinoma by baculovirus-mediated expression of long non-coding RNA PTENP1 and MicroRNA regulation. Biomaterials..

[29] Zhang Y, Xu C (2019). G allele of rs7853346 polymorphism in PTENP1 enhances the proliferation of multiple myeloma cancer stem cells by promoting the expression of PTENP1 and its downstream signaling molecules. J Cell Biochem.

[30] Gao L, Ren W, Zhang L, Li S, Kong X, Zhang H (2017). PTENp1, a natural sponge of miR-21, mediates PTEN expression to inhibit the proliferation of oral squamous cell carcinoma. Mol Carcinog.

[31] Yu G, Yao W, Gumireddy K, Li A, Wang J, Xiao W (2014). Pseudogene PTENP1 functions as a competing endogenous RNA to suppress clear-cell renal cell carcinoma progression. Mol Cancer Ther.

[32] Chen YX, Zhu DY, Gao J, Xu ZL, Tao SC, Yin WJ (2019). Diminished membrane recruitment of Akt is instrumental in alcohol-associated osteopenia via the PTEN/Akt/GSK‐3β/β‐catenin axis. FEBS J.

[33] Wang L, Zhang N, Wang Z, Ai D-m, Cao Z-y (2016). Pan H-p. Pseudogene PTENP1 functions as a competing endogenous RNA (ceRNA) to regulate PTEN expression by sponging miR-499-5p. Biochemistry (Moscow).

[34] Wang C-G, Wang L, Yang T, Su S-L, Hu Y-H, Zhong D (2020). Pseudogene PTENP1 sponges miR-214 to regulate the expression of PTEN to modulate osteoclast differentiation and attenuate osteoporosis. Cytotherapy.

[35] Liu Y, Liu L, Zhang J (2021). Protective role of matrine in sepsis-associated cardiac dysfunction through regulating the lncRNA PTENP1/miR-106b-5p axis. Biomed Pharmacother.

[36] Yuan M, Zhao S, Chen R, Wang G, Bie Y, Wu Q (2020). MicroRNA–138 inhibits tumor growth and enhances chemosensitivity in human cervical cancer by targeting H2AX. Exp Ther Med.

[37] Ge Y, He Y, Jiang M, Luo D, Huan X, Wang W (2017). Polymorphisms in lncRNA PTENP1 and the risk of gastric cancer in a Chinese population. Dis Markers.

[38] Yang S, Fu Z-z, Zhang Y-q, Fu B-h, Dong L-x (2021). Rs7853346 Polymorphism in lncRNA-PTENP1 and rs1799864 polymorphism in CCR2 are associated with radiotherapy-induced cognitive Impairment in subjects with glioma via regulating PTENP1/miR-19b/CCR2 signaling pathway. Biochem Genet.

[39] Huang J, Zheng Y, Xiao X, Liu C, Lin J, Zheng S (2020). A circulating long noncoding RNA panel serves as a diagnostic marker for hepatocellular carcinoma. Dis Markers.

[40] Xin C, Li J, Zhang Y, Yu Z (2018). Polymorphisms in lncRNA PTENP1 and the risk of oral squamous cell carcinoma in a Chinese population. Eur Rev Med Pharmacol Sci.

